# Staphylococcal Phenotypes Induced by Naturally Occurring and Synthetic Membrane-Interactive Polyphenolic β-Lactam Resistance Modifiers

**DOI:** 10.1371/journal.pone.0093830

**Published:** 2014-04-03

**Authors:** Lucia Palacios, Helena Rosado, Vicente Micol, Adriana E. Rosato, Patricia Bernal, Raquel Arroyo, Helen Grounds, James C. Anderson, Richard A. Stabler, Peter W. Taylor

**Affiliations:** 1 School of Pharmacy, University College London, London, United Kingdom; 2 Universidad Miguel Hernández, Elche, Alicante, Spain; 3 The Methodist Hospital Research Institute, Houston, Texas, United States of America; 4 Department of Chemistry, University College London, London, United Kingdom; 5 London School of Hygiene and Tropical Medicine, London, United Kingdom; National Institutes of Health, United States of America

## Abstract

Galloyl catechins, in particular (-)-epicatechin gallate (ECg), have the capacity to abrogate β-lactam resistance in methicillin-resistant strains of *Staphylococcus aureus* (MRSA); they also prevent biofilm formation, reduce the secretion of a large proportion of the exoproteome and induce profound changes to cell morphology. Current evidence suggests that these reversible phenotypic traits result from their intercalation into the bacterial cytoplasmic membrane. We have endeavoured to potentiate the capacity of ECg to modify the MRSA phenotype by stepwise removal of hydroxyl groups from the B-ring pharmacophore and the A:C fused ring system of the naturally occurring molecule. ECg binds rapidly to the membrane, inducing up-regulation of genes responsible for protection against cell wall stress and maintenance of membrane integrity and function. Studies with artificial membranes modelled on the lipid composition of the staphylococcal bilayer indicated that ECg adopts a position deep within the lipid palisade, eliciting major alterations in the thermotropic behaviour of the bilayer. The non-galloylated homolog (-)-epicatechin enhanced ECg-mediated effects by facilitating entry of ECg molecules into the membrane. ECg analogs with unnatural B-ring hydroxylation patterns induced higher levels of gene expression and more profound changes to MRSA membrane fluidity than ECg but adopted a more superficial location within the bilayer. ECg possessed a high affinity for the positively charged staphylococcal membrane and induced changes to the biophysical properties of the bilayer that are likely to account for its capacity to disperse the cell wall biosynthetic machinery responsible for β-lactam resistance. The ability to enhance these properties by chemical modification of ECg raises the possibility that more potent analogs could be developed for clinical evaluation.

## Introduction

There is an on-going need to develop new agents and provide new strategies for the treatment of hospital- and community-acquired infections caused by *Staphylococcus aureus*. This opportunistic pathogen has proven to be adept at acquiring genes encoding resistance mechanisms against front-line antibiotics and, in the absence of appropriate preventative measures, multi-drug-resistant forms such as methicillin-resistant *S. aureus* (MRSA) clones are able to disseminate at sometimes alarming rates amongst patients in healthcare facilities [1], [2]. Although it is becoming clear that the introduction of active surveillance followed by decolonization and contact isolation procedures can produce dramatic reductions in the incidence of hospital-acquired infections due to MRSA [3], [4], adverse infection and mortality rates [5] and high treatment costs [6] associated with MRSA infections indicate that the development of more effective therapeutic and preventative options remains a priority. In particular, novel modalities that reduce or abrogate the emergence of antibiotic resistance mechanisms are highly desirable [7], [8].

Members of the flavonoid group of polyphenolic secondary metabolites substantially modify the properties of pathogenic bacteria in ways that could benefit the infected patient: they have been shown, at least *in vitro*, to attenuate processes associated with bacterial pathogenicity, such as inhibition of quorum sensing signalling mechanisms and secretion of virulence effectors that include toxins and enzymes associated with bacterial defense against host factors [9]. Most importantly, some have the capacity to interfere with antibiotic resistance mechanisms, converting antibiotic resistant Gram-positive bacteria to a state of phenotypic susceptibility [8], [10], and raising the possibility that druggable versions of these molecules could be used therapeutically alongside conventional antibiotics whose utility has been compromised by the dissemination of resistance genes. Indeed, use of the highly successful combination of the β-lactamase inhibitor clavulanic acid and the β-lactam agent amoxicillin, marketed as Augmentin, is guided by such principles [11].

Galloyl catechins such as (-)-epicatechin gallate (ECg), (-)-epigallocatechin gallate (EGCg) and (-)-catechin gallate (Cg) are abundant components of the leaf of the green tea plant (*Camellia sinensis*). They have negligible antibacterial activity but show the capacity, at relatively low concentrations, to reduce penicillin-binding protein (PBP) 2a-mediated resistance to a wide range of β-lactam drugs [10], [12], [13]. These molecules scavenge free radicals and show a strong tendency to partition into model lipid bilayers comprising single phospholipid species such as phosphatidylglycerol (PG) and phosphatidylethanolamine (PE), penetrating deep into the hydrophobic core of the lipid palisade [14]. Their non-galloyl homologs (-)-epicatechin (EC), (-)-epigallocatechin (EGC) and (-)-catechin (C) interact more superficially with PC and PE bilayers, localizing close to the phospholipid-water interface [14], and they do not have the capacity to modulate β-lactam resistance in MRSA [12]. EC and EGC are, however, able to enhance the β-lactam-modifying potential of ECg and to increase the binding of ECg to staphylococcal cells [15]. Further, EC and other non-galloyl catechins markedly increase the quantities of EGCg and ECg that are incorporated into artificial lipid bilayers [16]. ECg has a higher affinity for membrane bilayers [14], [16], [17] and a greater capacity to modulate β-lactam resistance [12] than either EGCg or Cg, suggesting that a catechin-induced increase in the lipid order of the staphylococcal cytoplasmic membrane (CM), producing tightly packed and extended acyl chains in the bilayer [14], is the primary event determining increased β-lactam susceptibility. Support for this view comes from the complex changes to the staphylococcal phenotype which accompanies abrogation of resistance. These include a reduction in peptidoglycan cross-linking, impairment of the processing and *in situ* activity of cell wall autolysins, a thickened cell wall and poor separation of daughter cells following division [18], a large reduction in D-alanyl esterification of cell wall teichoic acid (WTA) [19], and loss of halotolerance [20]; there is a high probability that this phenotype is due to alteration of the biophysical properties and function of the CM.

MRSA strains are resistant to β-lactam antibiotics because they have acquired one of several allotypes of a mobile genetic element, the SCC*mec* cassette, which includes the *mecA* gene encoding the low-affinity penicillin-binding protein PBP2a [21]; this transpeptidase, which forms a functional complex with PBP2, allows peptidoglycan synthesis to continue after β-lactam-mediated acylation of native, membrane-localized PBPs [22]. Staphylococcal peptidoglycan synthesis is highly regulated and the CM-associated FtsZ-anchored biosynthetic machinery which in MRSA includes functional PBP2/2a complexes, is located predominantly at the division septum [23], facilitating orderly equatorial division in orthogonal planes [24]. In methicillin-susceptible staphylococci, but not in MRSA, PBP2 is delocalized from the septal membrane following exposure to β-lactam agents [23], [25]. Growth of MRSA in the presence of ECg elicits delocalization of PBP2 but not FtsZ even in the absence of the β-lactam agent oxacillin [26], providing strong evidence that ECg sensitizes MRSA strains by disrupting the septal peptidoglycan machinery following intercalation into the CM. In contrast to the zwitterionic and/or neutral surface charge of PC or PE bilayers, the staphylococcal CM is comprised of a complex asymmetric mixture of a number of lipids with different charge characteristics, predominantly phosphatidylglycerol (PG), PG modified by enzymatic transfer of a lysine residue (LPG) and cardiolipin (CL) [26]–[28]. No information is currently available on the capacity of ECg or other galloyl catechins to intercalate into the staphylococcal CM save that ECg distributes predominantly but not exclusively to the membrane fraction of mid-logarithmic bacteria [26].

**Figure 1:**
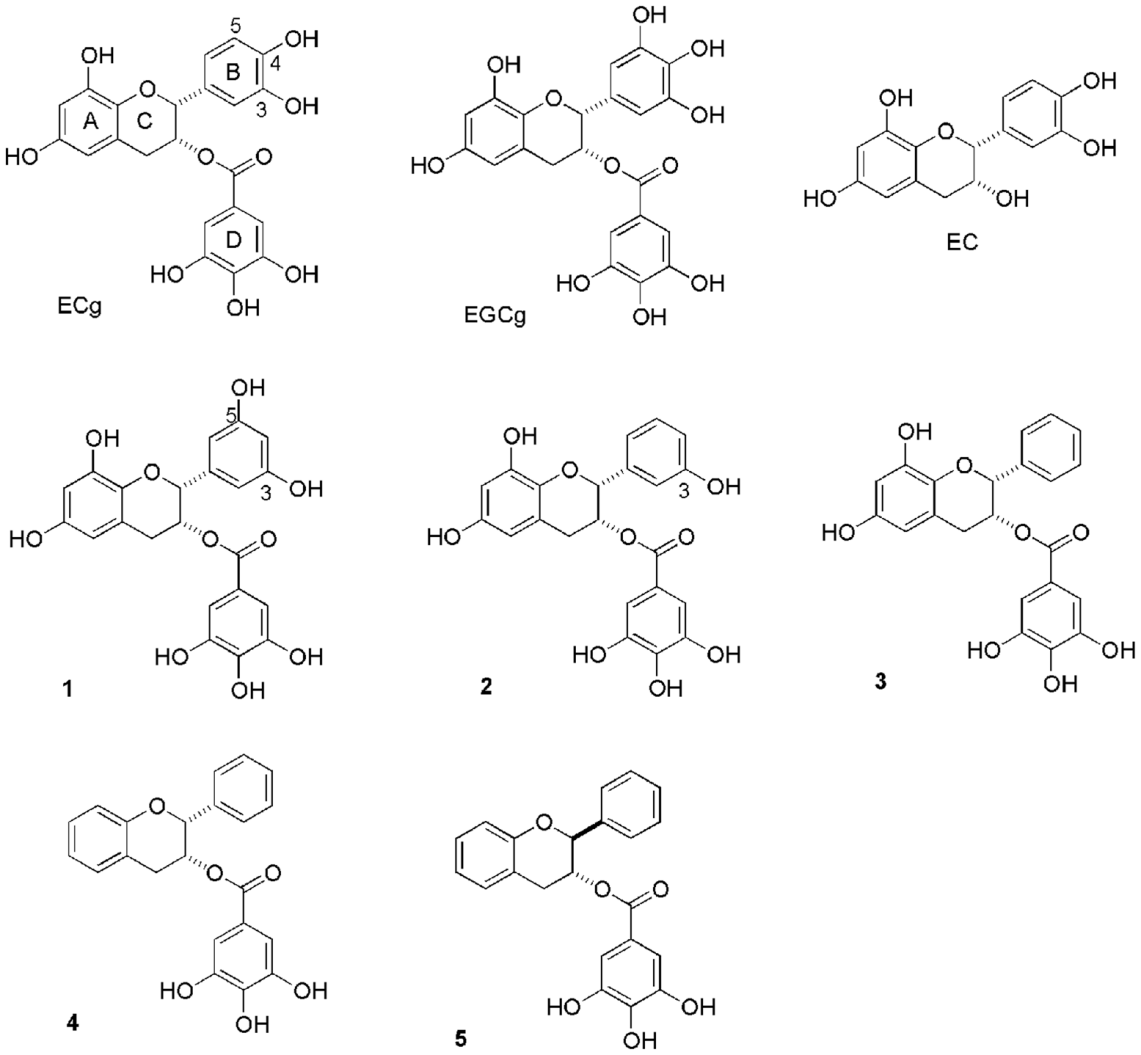
Structures of (-)-epicatechin gallate (ECg), (-)-epigallocatechin gallate (EGCg), (-)-epicatechin (EC), (-)-3,5-dihydroxy B-ring modified (-)-ECg (1), (-)-3-hydroxy B-ring modified (-)-ECg (2), (-) B-ring modified (-)-ECg (3), (-)- A,B-ring modified (-)-ECg (4) and A,B-ring modified (-)-Cg (5).

There is little doubt that ECg modifies the staphylococcal phenotype following interactions with the cell envelope. In common with cell wall- and CM-active antibiotics it invokes the cell wall stress stimulon [26], a set of genes up-regulated to preserve and repair a compromised cell wall or membrane [29], [30]. The relative affinity of galloyl catechins for lipid bilayers is dependent on their lipophilicity [31] and they appear not to gain entry to the cytoplasm of bacteria to any great extent [26]. ECg differs from EGCg only by the absence of a hydroxyl function at one of the *meta* positions on the B-ring (Fig. 1), suggesting that reducing the degree of hydroxylation or the position of hydroxyl groups on the B-ring pharmacophore may increase bilayer affinity, with consequent increases in bioactivity. We therefore synthesized a number of unnatural ECg analogs differing in B-ring hydroxylation and in hydroxyl substitution of the fused A-C-ring moiety [32], [33]. In this study, we investigated the capacity of natural and synthetic galloyl catechins, as well as combinations of galloyl and non-galloyl catechins, to interact with artificial LPG:PG:CL membrane bilayers, alter the biophysical properties of the staphylococcal CM *in situ* and modulate gene expression in MRSA. The data has shed light on the potential for creating therapeutic catechin combinations for modulation of staphylococcal β-lactam resistance.

## Materials and Methods

### Bacterial strains and reagents

Epidemic MRSA strain EMRSA-16 was isolated from a clinical sample obtained at the Royal Free Hospital, London. Minimum inhibitory concentration (MIC) was determined as described previously [12]. Bacteria were grown with aeration at 37°C in Mueller-Hinton (MH) broth (Oxoid Ltd., Basingstoke, UK), unless otherwise stated. The chloride salt of 1,2-dipalmitoyl-*sn*-glycero-3-[phospho-*rac*-3-(lysyl(1-glycerol))] (LPG), the sodium salt of 1,2-dipalmitoyl-*sn*-glycero-3-phospho-(1′-*rac*-glycerol) (DPPG) and the sodium salt of 1′,3′-bis[1,2-dimyristoyl-*sn*-glycero-3-phospho]-*sn*-glycerol (CL) were obtained from Avanti Polar Lipids (Birmingham, AL). Stock solutions of lipids were prepared in chloroform/methanol (1∶1) and stored at –20°C. The fluorescent probes 1,6-diphenyl-1,3,5-hexatriene (DPH) and 1-(4-trimethyl-ammonium phenyl)-6-phenyl-1,3,5-hexatriene (TMA-DPH) and the spin labels 5-doxyl-stearic acid (5-NS) and 16-doxyl-stearic acid (16-NS) were purchased from Molecular Probes (Eugene, OR). ECg, EGCg and EC were gifts from Mitsui Norin, Tokyo, Japan. (-)-3,5-dihydroxy B-ring modified (-)-ECg (**1**), (-)-3-hydroxy B-ring modified (-)-ECg (**2**), (-) B-ring modified (-)-ECg (**3**), (-)- A,B-ring modified (-)-ECg (**4**), and A,B-ring modified (-)-Cg (**5**) were synthesized as previously described [32], [33] with some modifications. Their structures are shown in Figure 1.

### Selection of daptomycin-resistant EMRSA-16

EMRSA-16 (10^9^ CFU/ml) was serially diluted in MH agar containing 50 μg/ml Ca^2+^ with increasing concentrations of daptomycin (DAP; 1×, 2×, 4×, and 8× DAP MICs; EMRSA-16 baseline DAP MIC: 0.5 μg/ml) as described [34]. Plates were incubated for 48 h at 35°C and colonies enumerated. Colonies isolated on DAP-containing agar plates were examined for DAP susceptibility. Stability of EMRSA-16DAP4 (obtained with 4 μg/ml DAP) was assessed by daily passage on antibiotic-free medium for 5 days.

### Mutational insertion inactivation of *mprF*

EMRSA-16 *mprF*-null mutants were constructed by integrational disruption through a single-crossover event. An 804-bp DNA fragment corresponding to the internal region of *mprF* was amplified by PCR using 5′-gcaatcacattgtatcgggag-3′ and 5′-cgggatccggtacaaaatagtacgcaa-3′ primers with chromosomal DNA from *S. aureus* RN4220 as template, and cloned into the *Sma*I site of pCK20. pCK20 containing the *E. coli* pUC19-derived origin and the chloramphenicol resistance gene was constructed by self-ligation of the *Afl*II-*Ava*II fragment of pND50 [35] to delete the *S. aureus* pUB110-derived origin. The resultant plasmid (pCK20-*mprF*) was electroporated into RN4220 and homologous recombinants selected on Luria-Bertani agar plates containing 12.5 μg/ml chloramphenicol and CK1001. EMRSA-16 *mprF* mutant was obtained by transducing CK1001 [36] into EMRSA-16 and EMRSA-16DAP4 by general transduction with 80α phage [37]. Southern blot hybridization was performed to ensure a single integration of the chromosomal *mprF* locus into EMRSA-16. Pulsed-field gel electrophoresis was performed to confirm identity between EMRSA-16, EMRSA-16Δ*mprF* and EMRSA-16Δ*mprF*DAP4.

### Cell membrane labelling and fluorescence anisotropy measurements

Membrane fluidity of mid-logarithmic EMRSA-16 was determined by measuring the fluorescence polarization of DPH inserted into the CM as previously described [38]. Fluorescence polarization was measured using a thermostat-controlled Perkin Elmer LS55 Luminescence Spectrometer. Excitation and emission wavelengths for DPH were 358 and 428 nm respectively. Data were analysed using FL WINLAB software program, version 4.00.02.

### Microarray Analysis

The BµG@S SAv1.1.0 microarray has been described elsewhere [39]; it contains PCR products representing all predicted open reading frames from the initial seven *S. aureus* genome sequencing projects. Total RNA extracts were prepared as described previously [40]. ECg was added to logarithmic cultures of EMRSA-16 at OD_600_ 0.28 or 0.33 and harvested after 1 h incubation at 37°C, corresponding to OD_600_ of 0.6–0.8 and 0.9–1.0 respectively. Hybridizations were performed as described [40]. Hybridization data was analyzed using an Affymetrix 428 scanner and the fluorescence intensity information was quantified using ImaGene version 5.1 (BioDiscovery, El Segundo, CA). The ImaGene data files were subjected to statistical analysis using the *Limma* software package for R [41], [42]. Briefly, background correction was performed; Loess normalization was applied to each print-tip group separately. The arrays were then normalized together by scaling the log ratios to ensure consistency between arrays. Ratios for each spot were calculated and an estimate made of the correlation between within-array spot replicates, under the assumption that the between-replicate correlation of each gene is common across all genes [42]. A moderated *t*-test was applied to each gene, where the variance was calculated as a combination of the variance for the gene, the pooled variance and the estimated spot replicate correlation. Each gene was then ranked according to the level of differential expression determined. The *P*-values were subsequently adjusted for multiple testing by False Discovery Rate. This test provides more sensitive analysis when compared with rank tests, as it allows for deviation between within-array replicate spots when estimating the accuracy of the data for each individual gene. Array design is available in BμG@Sbase (Accession No. A-BUGS-42; http://bugs.sgul.ac.uk/A-BUGS-42) and ArrayExpress (Accession No. A-BUGS-42). The modulation of selected genes by ECg was confirmed using qRT-PCR as described [36]; the primers used are shown in Table 1.

**Table 1:** Oligonucleotide primers used for qRT-PCR.

Gene	ORF	Forward primer	Reverse primer
***16S***	SARr001	GAC GGT CTT GCT GTC ACT TA	AGT TCC AGT GTG GCC GAT CA
***agrA***	SAR2126	ATG GAA ATT GCC CTC GCA AC	ACC AAC TGG GTC ATG CTT AC
***atl***	SAR1026	GTG GTT GGT CTA AGC GTA AG	CAA CGT GTA CCA GGT AAG TG
***blaZ***	SAR1831	CTT CAA CAC CTG CTG CTT TC	AGG TTC AGA TTG GCC CTT AG
***dltA***	SAR0894	AGC GTT AGT AAG CCG TTT CC	AGA TAA TCT TGC GCC TGG TC
***fmtA***	SAR1030	TAC ATG CGT GCG TGC TTT	GAT GGC TCA CCT TTG CCT TT
***isaA***	SAR2650	GCA GTG GCA TTA GGT GTA AC	CTG CTT CAT AGC TCC ATG AC
***lytM***	SAR0273	CCA GAC GCG AGC TAT TAT TC	GTC GCT TTA CTT GCT GAT CC
***mecA***	SAR0039	TGG CTA TCG TGT CAC AAT CG	GCA GTA CCT GAG CCA TAA TC
***mgrA***	SAR0739	GAA GCT GAA GCG ACT TTG TC	AGC ACT CGA TAC TGG TAC AG
***mprF***	SAR1372	TCA ACC GTA TGT CCC TTG TC	AGC CAC CAA AGC CTA CAA TC
***mreC***	SAR1729	CCC AAT CGT ACC AGC AAC AA	TTG GGC TGT CCA TAC GTT CA
***pbp1***	SAR1157	GCA GCC TAA ACG TGG TGA TG	GTT GGT CGC TGA CTG TAT GC
***pbp2***	SAR1461	GGT ACT TAC GGT GCT GAA AC	GGC TAT GTC CCA CAA ATG AG
***pbp3***	SAR1629	AAT GAG TCT GTG CCA AGA GG	AGC CGT ATC CAA CAT TTC CG
***pbp4***	SAR0652	GTA CAC GCG GAC TAT CCA AG	GTG CTA ATC CAG CGA CTA GG
***prsA***	SAR1932	CGC TAG TGC CAC AGA TTC	TCT TTA CCG CCG TAT TGC
***qoxA***	SAR1034	TTA CGA CCT CTG AAC GTA CC	CCC TAA AGA TCG TCC TGT TG
***qoxB***	SAR1033	AAA CGA CGT GGC ATA CCA TC	TAG CAA TGG CAT CTG CTG AC
***sgtB***	SAR1964	CTA CAC GCG ATA ATG TGG	GCA CAT CTC TGT CGC TAA
***vraA***	SAR0580	ACG CAA TTA CAG CAC TAC CC	GTA CGA CCG GAA CTT AAA GC
***vraR***	SAR1974	TCT GAG TCG TCG CTT CTA CA	GTG AAG GCG CTT CTG GTA AA
***vraS***	SAR1975	CGC ATT TCT AGC TGC GAA TC	GAC TAG CAC GAG AAC TTC AC
***vraT***	SAR1976	ACC GGC AAT ATA ACC TGC AC	ACA TGG AAT TGG CGA CCT AC
***vraX***	SAR0584	ATC AAC ATG AAG GCG CAC CA	CGT CTT GTA ATA AAG AGA GC

### Determination of cell surface charge

Staphylococcal surface charge was determined by cytochrome *c* binding, essentially according to Mukhopadhyay et al [28]. Cationic equine heart-derived cytochrome *c* binds to cells in proportion to the net negative surface charge. Mid-logarithmic phase cells were washed twice in 20 mM MOPS pH 7 and suspended in the same buffer to a final OD_600 nm_ of 7. The suspension was incubated with 0.5 mg/ml of cytochrome *c* for 10 minutes at room temperature and the supernatant recovered by centrifugation. A MOPS buffer control without cells was treated in the same manner. The remaining (unbound) cytochrome *c* in the supernatant was quantified spectrophotometrically at 530 nm (the absorption maximum of the prosthetic group) and compared to the buffer control (equivalent to 100% unbound). Each experiment was repeated a minimum of three times on different days.

### Differential scanning calorimetry (DSC)

Chloroform/methanol solutions containing 3 μmol of lipid mixture and appropriate amounts of catechin were dried under a stream of oxygen-free N_2_ and solvent completely removed under high vacuum for at least 3 h. Multilamellar vesicles (MLVs) were formed by incubating the dried lipid in 2 ml of suspension buffer (20 mM sodium phosphate, 130 mM NaCl, pH 7.4) for 15 min at a temperature above the gel to liquid-crystalline phase transition of the lipid (approx. 55°C) with occasional vigorous vortexing followed by three freeze-thaw cycles. Thermograms were recorded on a high-resolution Microcal MC-2 differential scanning microcalorimeter equipped with a DA-2 digital interface and data acquisition utility for automatic data collection as previously described [14], [43].

### Steady-state fluorescence anisotropy of MLVs

DPH or its cationic derivative TMA-DPH was dissolved in *N*, *N*′-dimethylformamide and added to lipid dispersions prior to the formation of MLVs, as described in the preceding section, to obtain a probe:lipid molar ratio of 1∶500. MLVs were incubated for 1 h above the gel to liquid-crystalline phase transition temperature and the steady-state fluorescence anisotropy (<r>) of DPH measured with a Varian Clary Eclipse spectrofluorimeter as previously described [43] at a constant rate of 0.25°C/min over the range 20 - 60°C. Samples were stabilized at the recording temperature and measurements obtained under continuous mixing. <r> is defined as:
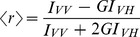



*G*-factor, accounting for differential polarization sensitivity, was determined by measurement of the polarized components of probe fluorescence with horizontally polarized excitation (*G* =  *I*_HV_/*I*_HH_). The vertically- and horizontally-polarized emission intensities, elicited by vertically-polarized excitation, were corrected for background scattering by subtraction of the corresponding polarized intensities of a phospholipid preparation lacking probes. Samples were excited at 360 nm and polarised emission was recorded at 430 nm.

### Quenching of catechin fluorescence in lipid vesicles

Differential quenching data using 5-NS and 16-NS spin probes were analyzed by Stern–Volmer plots of *I*_0_/*I* versus [*Q*]_L_, where *I*_0_ and *I* are, respectively, the fluorescence intensities of the catechin in the absence and presence of the quencher and [*Q*]_L_ is the quencher concentration in the phospholipid phase given by:
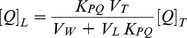



*K*_PQ_  =  [*Q*]_L_/[*Q*]_W_ is the partition coefficient of the quencher between the phospholipid phase and the aqueous phase, [*Q*]_T_ the concentration of the quencher in the total volume *V*_T_ (V_T_  =  *V*_L_ + *V*_W_) and *V*_L_ and *V*_W_ are, respectively, the volumes of the lipid and aqueous phases. For 5-NS and 16-NS, values for *K*_PQ_ in the fluid phase are 89,000 and 9,730 respectively [44]. Experiments were carried out at 45°C in large unilamellar vesicles (LUVs) containing catechins at saturating-lipid conditions by the addition of aliquots from a 1 mM ethanolic solution of either 16-NS or 5-NS to the vesicle suspension. Samples were excited at 335 nm and fluorescence emission was recorded at 445 nm. Measurements were taken immediately after preparation [43].

The hydrophilic quencher acrylamide was used to determine the presence of catechin molecules accessible to the aqueous phase and to localize catechins in phospholipid bilayers [14]. LUVs containing appropriate amounts of catechin were incubated for 15 min at 55°C. Catechin quenching was then undertaken in the absence and presence of the vesicles under lipid saturating conditions by addition of acrylamide from a 4 M acrylamide aqueous solution to catechin-vesicle or catechin-buffer preparations. Data were analysed by modified Stern-Volmer plots that account for both collisional and static quenching modes [45].

### Determination of partition of catechins into MLVs

Catechin-containing MLVs were equilibrated by incubation for 60 min at 45°C and centrifuged for 20 min at 5,000 *g*. The catechin content of supernatants and precipitated fractions was determined by HPLC: 100 μl aliquots in chloroform-methanol solution were separated by reverse phase chromatography using a Merck LiChrospher 100 RP-18 column as described previously [46]. Compound identification was achieved after diode-array detection by comparison of retention time and UV spectra of peaks with standards. Quantitative evaluation of catechins was performed by means of a six-point regression curve (r2>0.996) in the range 0.25–100 μg/ml using external catechin standards. The phospholipid-water partition coefficient *K*_P_ was defined as the amount of catechin found in precipitated lipid vesicles divided by that in the supernatant of the vesicle preparation.

## Results

### Effect of catechins on antibiotic resistance

Incorporation of 12.5 μg/ml ECg into the assay reduced the MIC for oxacillin of EMRSA-16 from 512 to <1 μg/ml (*n* = 3). EC had no capacity to modify oxacillin resistance in EMRSA-16 but markedly enhanced the reduction of oxacillin resistance by ECg: for example, 3.12 μg/ml ECg reduced the MIC to 128 μg/ml but addition of 6.25 μg/ml EC to the assay yielded a MIC of 8 μg/ml. This enhancement was seen over a broad range of ECg and EC concentrations in various combinations [14]. ECg and analogs **1** and **2** possessed very weak intrinsic anti-staphylococcal activity, with MIC values of 128 μg/ml; in oxacillin resistance reduction assays, **1** and **2** at a concentration of 12.5 μg/ml were indistinguishable from ECg, modulating susceptibility from 512 to <1 μg/ml. The B-ring phenyl analog **3** produced a more pronounced (64 μg/ml), albeit still weak, anti-staphylococcal effect but displayed an enhanced resistance reduction capacity, modulating the EMRSA-16 MIC from 512 to 0.25 μg/ml at a concentration of 12.5 μg/ml. Deletion of all hydroxyl moieties on the A-C- and B-rings resulted in overtly antibacterial compounds. MICs of **4** and **5** were determined to be 16 μg/ml; as such, their oxacillin resistance reducing capacity could not be examined at our standard concentration of 12.5 μg/ml but at 6.25 μg/ml they reduced the MIC of EMRSA-16 to 16 and 4 μg/ml respectively, significantly less than compound **3** (2 μg/ml) at the same concentration.

### Changes in EMRSA-16 gene expression induced by catechins

We previously determined that growth to mid-logarithmic phase for 4 h in the presence of 12.5 μg/ml ECg led to ≥2-fold up-regulation of genes associated with the staphylococcal cell wall stress stimulon [26]. However, exposure to ECg for this period of time enables the bacterial cell to substantially reconfigure the CM by increasing the proportion of branched chain fatty acids in the bilayer, leading to a fluid structure that compensates for the initial increased rigidity imposed by the rapid intercalation of the galloyl catechin into the membrane [26]. Thus, at this time point, the transcriptomic response is unlikely to reflect the cellular response to the initial insult (incorporation of ECg into the bilayer leading to markedly decreased fluidity), an event that occurs immediately after exposure to the compound [26]. Recent work has shown that the induction kinetics of the cell wall stress stimulon are strongly dependent on the nature of the inducing agent and that induction occurs within the first two division cycles and peaks within the first 2 h of exposure [47]. We therefore determined the capacity of ECg, ECg analogs and the ECg/EC combination to induce the cell wall stress stimulon transcriptomic response 1 h after addition to early logarithmic phase cultures of EMRSA-16. This time point coincides with the induction maximum as determined by qRT-PCR using probes for two key cell wall stress stimulon genes and before ECg-induced reorganisation of the CM (data not shown); it also permits better comparison with gene transcription studies undertaken to determine the *S. aureus* response to β-lactam antibiotics [48] and vancomycin [49], with 60 and 30 min exposure respectively. In addition, ECg-treated cells exposed to oxacillin retain viability at this time interval [26].

ECg (12.5 μg/ml), analogs **1**, **2**, **3** (12.5 μg/ml) and **5** (12.5 μg/ml) were employed to examine transcriptomic responses. EC was used at a concentration of 12.5 μg/ml. Addition of ECg at OD_600_ 0.28 and 0.33 produced comparable gene expression profiles. Exposure to ECg for 1 h was comparable to 4 h exposure to ECg [26] in that genes of the cell wall stress stimulon (for example, *vraR*, *vraS*, *vraX*, *lytM* and *tcaA*) were significantly up-regulated, in the case of *vraX* massively so (82.2-fold). A significant number of other genes that respond to the *vraSR* two-component sensor/regulator sentinel of cell wall stress [50] were also up-regulated by ECg: these included *prsA* (2.6-fold), encoding a facilitator of protein secretion and extra-cellular folding [51], and the cell surface modulator gene *msrR* (6.7-fold) [52]. Genes involved in β-lactamase expression (*blaZ*, 18-fold; *blaR1*, 9.4-fold) and those encoding proteins which influence the level of *S. aureus* methicillin resistance (*mecA*, 4.4-fold; *mecI*, 2.9-fold; *femA*, 2.1-fold; *femB*, 2.1-fold) were also up-regulated, as were *tagA* (2.2-fold) and *tagH* (2.2-fold) with roles in teichoic acid synthesis and genes encoding penicillin binding proteins involved in peptidoglycan biosynthesis (*pbp2*, 2.8-fold; *pbp4*, 3.0-fold) and peptidoglycan autolytic modelling (*isaA*, 12.7-fold) [53]. It is noteworthy that genes encoding the four proteins for D-alanyl esterification of WTA were up-regulated (*dltA* 3.7-fold; *dltB* 2.5-fold; *dltC* 3.2-fold; *dltD* 3.1-fold), as we have previously determined that exposure of EMRSA-16 to ECg led to a large reduction in D-alanine content of WTA [19]. ECg also reduces the capacity of EMRSA-16 to attach lysine residues to PG lipid head groups and the gene encoding the phosphatidylglycerol lysyltransferase responsible for this function, *mprF*, was up-regulated by ECg.

EC had minimal impact on gene transcription, with only one gene of unknown function up-regulated (at the early ECg addition point) and no significant gene down-regulation. The pattern of modulation of gene expression when ECg and EC were combined was similar to that for ECg alone. Compounds **1**, **2** and **3** induced gene transcription profiles that were broadly similar to those for ECg but the more overtly antibacterial analogue **5** elicited a markedly different response at 0.5 MIC: very few genes up-regulated by ECg were induced and there was down-regulation of a large number of ribosomal, metabolic, shock, virulence and transport-related genes. We therefore selected 24 genes (Table 2), on the basis of their relevance to the perceived mode of β-lactam resistance modulation of galloyl catechins [8], [18], [19], [26], [33], in order to more closely examine by qRT-PCR the relative impact of ECg, ECg/EC and analogs **1**, **2**, **3** and **5** on EMRSA-16.

**Table 2:** qRT-PCR^a^ determination of changes to EMRSA-16 gene expression following exposure to ECg, EC and structural analogs of ECg.

Gene^b^	ORF	Function	ECg	EC	ECg/EC	1^c^	2	3	5
*dltA*	SAR0894	D-alanine-D-alanyl carrier protein ligase	1.58*^d^	1.00	1.09	1.47	1.32	1.47	0.12*
*mecA/pbp2a*	SAR0039	penicillin binding protein 2 prime	1.36*	1.18	1.15	1.37*	1.49*	0.88	0.23*
*pbp1*	SAR1157	penicillin binding protein 1	0.75	0.87	0.87	0.86	0.89	1.54*	0.25*
*pbp2*	SAR1461	penicillin-binding protein 2	1.16	1.07	0.89	2.49*	2.42*	4.01*	0.27*
*pbp3/pbpF*	SAR1629	penicillin-binding protein PBP2B	0.80	0.94	0.67	0.84	0.60*	0.81	0.13*
*pbp4*	SAR0652	penicillin-binding protein 4	0.76*	1.03	0.71*	0.67*	0.55*	0.69	0.14*
*prsA*	SAR1932	foldase protein PrsA	3.44*	1.00	2.77*	4.57*	4.96*	7.85*	1.04
*sgtB/mgt*	SAR1964	monofunctional glycosyltransferase	2.61*	1.20	2.19*	4.25*	6.24*	5.03*	1.91*
*vraR/yvqC*	SAR1974	two-component response regulator	2.21*	1.63	1.72*	3.66*	1.95*	5.69*	0.86
*vraS/yvqE*	SAR1975	two-component sensor histidine kinase	2.20*	0.65	1.51*	4.95*	2.41*	11.01*	0.92
*vraT/yvqF*	SAR1976	response regulator	2.93*	0.97	2.24*	3.10*	2.90*	5.81*	0.91
*mreC*	SAR1729	cell shape determining protein MreC	1.08	1.11	0.85	0.65*	0.45*	0.99	0.55*
*mprF/fmtC*	SAR1372	phosphatidylglycerol lysyltransferase	2.23*	1.59	2.26*	1.49*	1.89*	1.81*	0.48*
*agrA*	SAR2126	accessory gene regulator protein A	0.28*	1.25	0.40*	0.18*	0.15*	0.12*	0.02*
*blaZ*	SAR1831	β-lactamase	1.86	0.94	1.28	3.35*	3.17*	3.79*	0.33*
*atl/nag*	SAR1026	bifunctional autolysin	0.74	1.27	0.81	0.91	0.70	0.71	0.05*
*fmtA/fmt*	SAR1030	protein FmtA	1.81*	1.36	1.56	1.44	0.90	0.90	0.08*
*isaA*	SAR2650	immunodominant antigen A	2.41*	1.08	1.57	1.42	1.18	1.25	0.02*
*lytM*	SAR0273	glycyl-glycine endopeptidase LytM	3.16*	1.40	2.86*	3.60*	5.63*	4.87*	0.04*
*mgrA/nor*	SAR0739	HTH-type transcriptional regulator MgrA	1.11	1.79	0.99	0.57*	0.44*	0.45*	0.11*
*vraA*	SAR0580	long chain fatty acid-CoA ligase VraA	0.22*	0.50*	0.75*	0.45*	0.26*	0.37*	0.75
*qoxA/cydA*	SAR1034	quinol oxidase subunit 2	0.43*	0.43*	0.25*	0.31*	0.23*	0.14*	0.03*
*qoxB/cydB*	SAR1033	quinol oxidase subunit 1	0.43*	0.43*	0.35*	0.34*	0.15*	0.18*	0.04*
*vraX*	SAR0584	protein VraX	17.46*	1.09	14.39*	34.77*	40.29*	62.35*	7.31*

a Quantitative reverse transcription-polymerase chain reaction.

b MRSA252 ORF identifiers for the BµG@S SAv1.1.0 microarray used in this study (39).

c Refer to ECg analogs described in Fig. 1.

d Denotes statistical significance p<0.05 (Student's *t* test, *n* = 6 in duplicate).

The data emphasised the prominent up-regulation of *vraX* by ECg and its structural analogs. Although EC facilitates increased binding of ECg to staphylococcal cells [15], it reduced the capacity of ECg to modulate transcription of the selected genes in almost all cases. Compound **1**, with unnatural B-ring dihydroxylation and possessing comparable antibacterial and resistance modifying properties to ECg, up-regulated genes of the cell wall stress stimulon, especially *vraX*, to a significantly greater extent than ECg, indicating that it may bind more avidly to the CM. It also showed enhanced up-regulation of *blaZ* and *prsA*; up-regulation of *mprF*, although significant, was less than for ECg. The B-ring-modified analogs **2** and **3** were designed to penetrate the lipid bilayer to a greater extent than the natural compound and elicited further incremental up-regulation of the cell wall stress stimulon. In support of data showing that galloyl catechins reduce expression of staphylococcal virulence effectors [8], [54], all weakly antibacterial, β-lactam-resistance-modifying compounds in the series (ECg, **1**-**3**) down-regulated the pivotal accessory gene regulator *agr* responsible for control of expression of *S. aureus* toxins and virulence-related exoenzymes [55]. ECg down-regulated some metabolic genes linked to *graSR* regulation of virulence, the stress response and cell wall signal transduction [56], including *qoxA* and *qoxB*, and these genes were similarly modulated by compounds **1**-**3**. Analogs **4** and **5**, lacking hydroxyl functions on the A- and B-rings, both possessed significant antibacterial activity but **5** enhanced β-lactam susceptibility to a greater extent than **4**; consequently only **5** was investigated with respect to gene modulation by qRT-PCR. The sub-inhibitory concentration employed elicited a markedly different response in comparison to ECg, with down-regulation of the large majority of genes in Table 2, suggesting that this compound has the capacity to induce lethal damage to the membrane. Interestingly, *vraX* was an exception with 7.3-fold up-regulation. Microarray data have been deposited in BμG@Sbase (http://bugs.sgul.ac.uk/E-BUGS-153) and ArrayExpress (accession number E-BUGS-153).

### Effect of deletion of *mprF* on the EMRSA-16 phenotype

**Figure 2:**
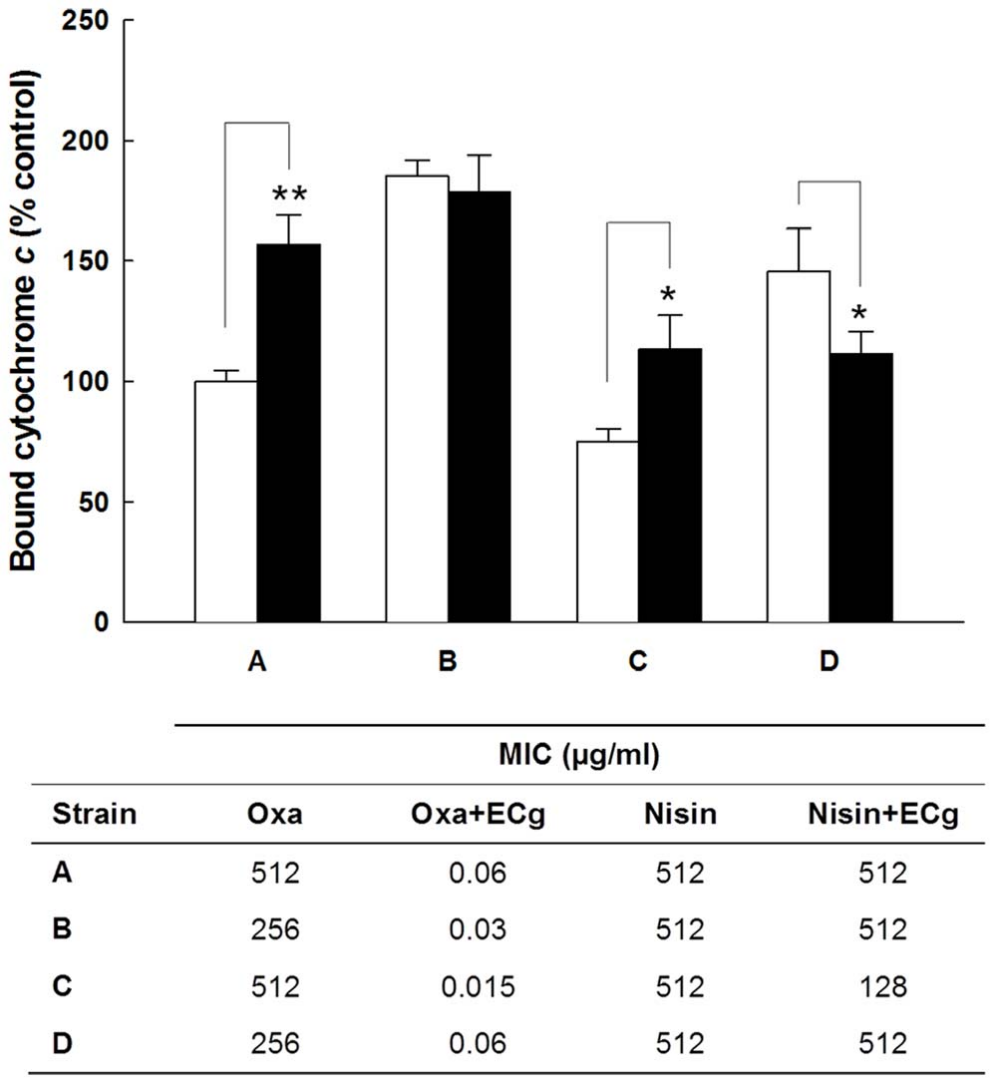
Phenotypic changes induced by ECg. Cytochrome *c* binding affinity and antibiotic susceptibility of *S. aureus* after exposure to ECg (▪) compared to untreated bacteria (□). Mid-logarithmic cultures of EMRSA-16 (A), EMRSA-16Δ*mprF* (B), EMRSA-16DAP4 (C) and EMRSA-16Δ*mprF*DAP4 (D) were exposed to ECg (12.5 μg/ml) for 4 h, the cells washed, incubated with cytochrome *c* (0.5 mg/ml) for 10 min and supernatants assayed spectrophotometrically at 530 nm (*n* = ≥4, ±1 SD). *p<0.001; **p<0.0001. For determination of minimum inhibitory concentration (MIC), ECg (12.5 μg/ml) was incorporated into the standard assay using MH broth containing 2% w/v NaCl (*n* = 4, ±1 SD).

Exposure of EMRSA-16 to ECg reduced the *mprF*-mediated attachment of lysine residues to PG head groups by almost 50% [26] and the β-lactam resistance modifiers employed here induced up-regulation of *mprF* (Table 2). To investigate if the *mprF* gene product was directly involved in ECg-mediated drug sensitisation, we deleted the gene in EMRSA-16 and determined its susceptibility to various agents in the presence and absence of ECg. EMRSA-16 is susceptible to vancomycin (MIC 0.5-1 μg/ml) and daptomycin (1 μg/ml) but resistant to nisin. EMRSA-16Δ*mprF* was marginally more susceptible to oxacillin (128 μg/ml compared to 256 μg/ml) than the parent strain and exposure to ECg led to a comparable reduction in oxacillin resistance (Figure 2), indicating that abrogation of lysine conjugation to PG does not account for β-lactam sensitisation. Binding of ECg to EMRSA-16 led to a large increase in the negative charge of the bacterial surface; however, exposure of EMRSA-16Δ*mprF* to ECg did not result in any alteration of surface charge, highlighting the large contribution to the overall surface charge profile from lysine residues. It is generally recognised that lysinylation of PG in the outer leaflet of the staphylococcal CM confers resistance to antimicrobial peptides, including nisin, through electrostatic repulsion [57], [58], but loss of MprF-mediated lysinylation had no impact on the nisin susceptibility of EMRSA-16 (Figure 2). As mutations conferring daptomycin resistance may result in decreased CM fluidity, increased synthesis and expression of LPG and increased flipping of LPG to the outer leaflet of the CM [59], an *in vitro*-selected EMRSA-16 mutant (EMRSA-16DAP4) resistant to daptomycin at 4 μg/ml was examined. EMRSA-16DAP4 bound significantly less cytochrome *c* (65%) than the parent strain, indicative of increased expression of *mprF* [59], and ECg induced a comparable increase in the negative surface charge. These alterations were not accompanied by changes in oxacillin or nisin susceptibility, although ECg sensitised EMRSA-16DAP4 to oxacillin to a greater extent than to EMRSA-16 and also induced a significant reduction in resistance to nisin compared to the parent strain (Figure 2). Inactivation of *mprF* in EMRSA-16DAP4 did not significantly alter susceptibility to the two antibiotics; in contrast to EMRSA-16DAP4, the net surface charge of EMRSA-16Δ*mprF*DAP4 was rendered more positive following exposure to ECg (Figure 2).

### Impact on CM fluidity

**Figure 3:**
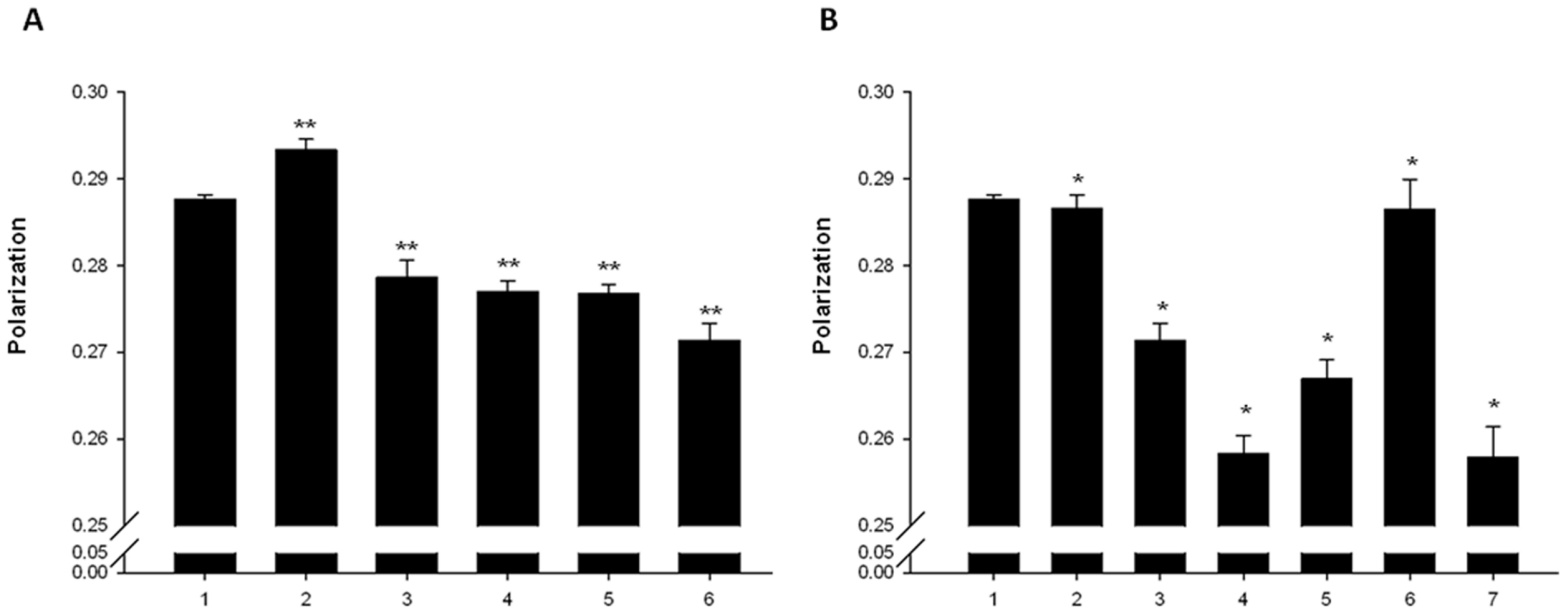
Effect of catechins on membrane fluidity of EMRSA-16. The fluorescent probe DPH was incorporated into the CM as described under “Experimental Procedures”. *A*, fluorescence polarization (arbitrary units) before addition of ECg (*bar 1*), immediately after addition of 12.5 μg/ml ECg (*bar 2*) and after 1 (*bar 3*), 2 (*bar 4*), 3 (*bar 5*) and 4 h (*bar 6*) incubation at 37°C. *B*, fluorescence polarization after 4 h incubation with EC (*bar 2*), ECg (*bar 3*), ECg + EC (*bar 4*), compound 1 (*bar 5*), compound 2 (*bar 6*) and compound 3 (*bar 7*). Control (*bar 1*) before addition of ECg (n = ≥11, ±1 SD). *, p<0.01; **, p<0.001. All values compared to control (*bar 1*).

We measured the impact of ECg, EC/ECg and analogs **1**, **2** and **3** on membrane fluidity using DPH; the relative hydrophobicity of **4** and **5** [33] rendered them unsuitable for CM intercalation experiments. DPH, a hydrophobic fluorescent probe, associates with the lipophilic tails of phospholipids without disturbing bilayer structure [60]. A reduction in the freedom of rotation of the probe will be reflected in increased emission of light in the same plane as the excited light and a reduction in emission of light at 90° to the plane of excitation, reflecting an inverse relationship between the emission of polarized light and the fluidity of the membrane. As exposure of *S. aureus* to ECg induces a reconfiguration of the composition and biophysical properties of the CM [26], we determined DPH polarization over a 4 h period of incubation with ECg (Figure 3A). When ECg was added to DPH-loaded mid-logarithmic cultures of EMRSA-16, there was an immediate, significant (p<0.001) increase in membrane probe polarization, indicating a reduction in fluidity. By 1 h, DPH polarization had decreased significantly (p<0.001) to below the level of the control and the fluidity of the bilayer continued to increase over the 4 h incubation period. Changes to CM fluidity induced by EGCg at 0 and 4 h were comparable to the values obtained for ECg (data not shown). Incubation with EC for 4 h produced a small but significant (p<0.01) increase in EMRSA-16 CM fluidity; the combination of EC and ECg (both 12.5 μg/ml) elicited a significantly (p<0.01) larger increase in fluidity at 4 h compared to ECg (Figure 3B). Surprisingly, compound **2**, with reduced B-ring hydroxylation, did not induce a higher degree of CM fluidity than EC after 4 h incubation but **1** and **3** produced a more fluid membrane at this time point (Figure 3B), suggesting a greater degree of CM perturbation than the natural product for these two compounds.

### Catechin-mediated effects on model staphylococcal membranes

Significant insights into the interactions of lipid bilayers and green tea polyphenols have been gained from biophysical studies of the incorporation of catechins into model membranes [14], [61], [62]. However, such work has invariably been undertaken using membranes composed of single phospholipids such as PE and PC, sometimes uncovering relationships restricted to specific lipid species such as PE [14] that may not be present in significant quantities in staphylococcal bilayers. We therefore studied the incorporation of catechins into MLVs composed of the three main phospholipids found in the CM of EMRSA-16 (LPG:PG:CL) in the ratios (53∶43.5∶3.5) determined in an earlier study [26], with the PG component represented by DPPG.

**Figure 4:**
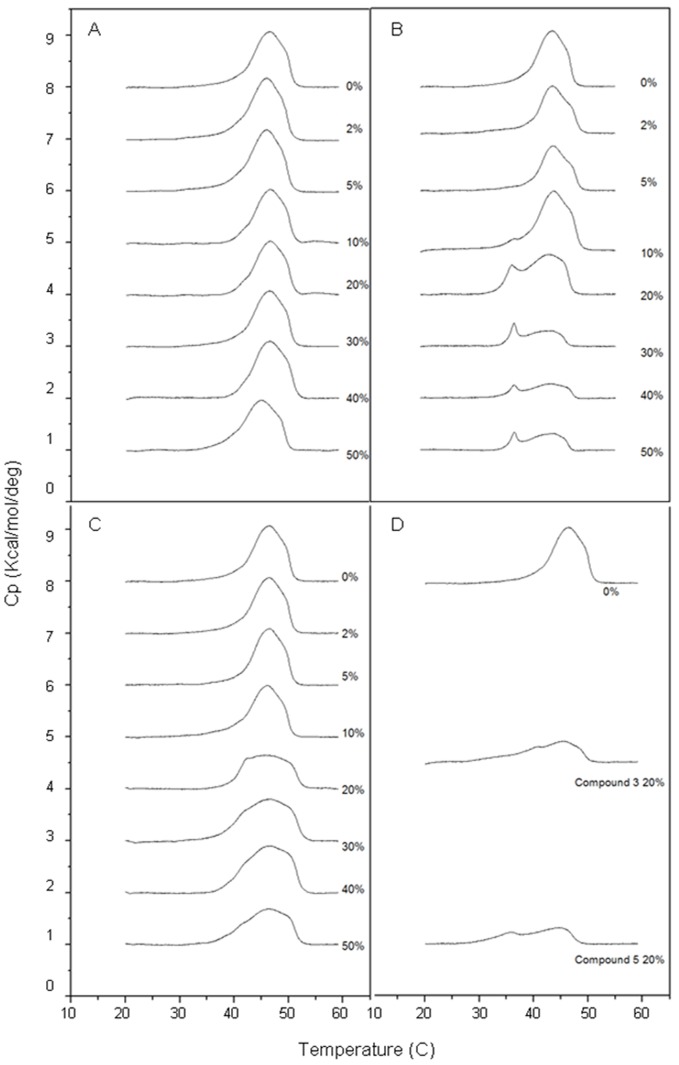
Impact of catechins on the thermotropic behavior of model membranes. Differential scanning calorimetry profiles of multi-lamellar LPG:PG:CL vesicles containing different concentrations of (A) EC, (B) ECg, (C) EC+ECg, (D) compounds **3** and **5** at a concentration of 20% total bilayer mass.

The impact of incorporation of catechins on the thermotropic behavior of LPG:PG:CL synthetic membranes was examined by DSC. The DSC profile of catechin-free membranes showed a broad and complex phase transition, with a main peak and a shoulder, most likely corresponding to phospholipid domains enriched for LPG or PG (Figure 4). Incorporation of increasing amounts of EC did not induce significant changes to the DSC profile of the phospholipid mixture; a high proportion (≥50 mol%) of EC led to a small shift of the main transition to a lower temperature (Figure 4A). In contrast, at concentrations of 10 mol% and above, ECg promoted significant changes in the properties of the bilayer with broadening of the transition and emergence of a narrow transition peak at ∼39°C, indicating the presence of a phospholipid domain with strong hydrophobic interactions (Figure 4B). The fluidus line of the lipid mixture remained practically horizontal over the range of ECg concentrations employed, indicating a high degree of component immiscibility, with phospholipid-catechin domains coexisting with free phospholipid. In contrast, the solidus line of this transition decreased gradually with increasing ECg concentration, indicating partial mixing of catechin and phospholipid. When equimolar concentrations of EC and ECg were incorporated into LPG:PG:CL bilayers, a broadening of the transition peak above 30 mol% was noted, but the peak was attenuated in comparison to samples containing only ECg. Compounds **3** and **5** were also examined at a molar percentage compared to total phospholipid of 20 mol%. In comparison to ECg at this concentration, both broadened and shifted the phase transition to a lower temperature, with **5** showing a more pronounced effect (Figure 4D) which could be replicated in bilayers composed of only PG or only LPG (data not shown), indicating that they interact with both phospholipids in LPG:PG:CL bilayers.

**Figure 5:**
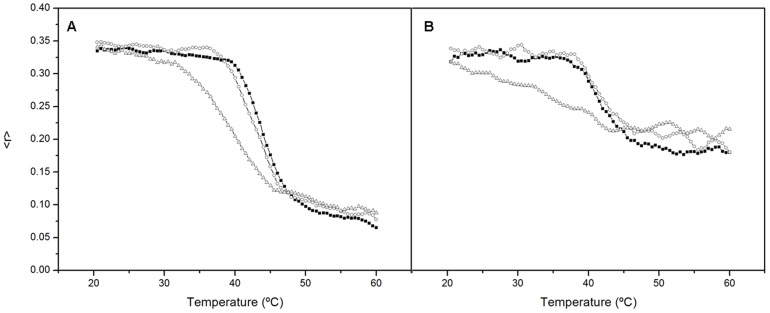
Impact of catechins on anisotropy of model membranes. Temperature variation of probe fluorescence anisotropy for DPH (A) and TMA-DPH (B) incorporated into LPG:PG:CL vesicles (•), and LPG:PG:CL vesicles containing either 20 mol% compound **3** (o), or 20 mol% compound **5** (◊). The DPH probe is located deep within the bilayer, whereas the TMA-DPH probe adopts a more superficial bilayer location. Compound **3** has almost no effect on anisotropy, but compound **5** elicits a major shift in the transition temperature and decreases anisotropy values in the gel phase and through the transition, indicating a decrease in lipid order and an increase in bilayer fluidity in comparison to compound **3** and vesicles alone.

The effect of incorporation of catechins on the phospholipid order of the synthetic membranes was also investigated by steady-state fluorescence anisotropy. Variations in the anisotropy of membrane-incorporated DPH and TMA-DPH fluorescence probes were recorded in the presence of increasing concentrations of catechins at temperatures below and above the gel to liquid-crystalline phase transition. The fluorophores of TMA-DPH and DPH are located approximately 12 and 7.8 Å from the center of the bilayer respectively [63], [64]. The presence of charged groups in TMA-DPH fixes its position in the membrane to a shallower location compared to the parental DPH probe [65], [66]. Neither EC nor ECg had a significant impact on the anisotropy values of DPH incorporated into the model bilayer at concentrations up to 40 mol%; an observed increase of the instability anisotropy signal was likely to be due to a higher degree of probe movement within the membrane (data not shown). In contrast, intercalation of ECg or EC, either alone or in combination, induced a small increase in anisotropy of TMA-DPH incorporated into the gel phase, indicating a mild decrease in membrane fluidity (data not shown). However, there was no evidence for a relationship between catechin concentration and anisotropic response and the effect was only observed at high catechin concentrations (>20 mol%). Compounds **3** and **5** both impacted on the anisotropy of DPH and TMA-DPH to a greater extent than the naturally occurring molecules and **5** showed a much stronger effect on the anisotropy of both membrane-incorporated probes than **3** (Figure 5). In agreement with DSC data, compound **5** shifted the transition temperature of LPG:PG:CL bilayers to a lower temperature and increased the anisotropy of DPH in the fluid phase, indicating decreased mobility of phospholipid acyl chains within this phase. A similar effect has been reported for ECg and diterpenes incorporated into PC vesicles [14], [66]. The impact of **5** on TMA-DPH anisotropy was more profound with respect to increased anisotropy values in the fluid phase and decreased anisotropy in the gel phase, indicating that this ECg analog elicited a more pronounced effect on phospholipid order within more superficial domains of the membrane than on the interior of the phospholipid palisade.

We previously estimated the phospholipid/water partition coefficient of catechins and other compounds by determining the variation of their intrinsic fluorescence following incorporation into lipid vesicles comprised of single phospholipid species [14], [43]. Here, we applied the same approach, but it proved difficult to obtain a stable catechin fluorescence signal, in all likelihood due to the complexity of vesicle composition and partition of catechins into heterogeneous phospholipid domains. We therefore determined the affinity of catechins by incubation with LPG:PG:CL membranes, centrifugation and HPLC quantification of catechins in pellets and supernatants. ECg showed a higher affinity for these bilayers (K_p_ 1.32±0.75) than EC (K_p_ 0.58±0.07). When ECg and EC were combined in a 1∶1 molar ratio, the affinity of both compounds for the model membranes was considerably enhanced (K_p_ ECg = 2.13±1.03; K_p_ EC: 1.75±0.45), in line with their synergistic impact on β-lactam sensitization.

**Figure 6:**
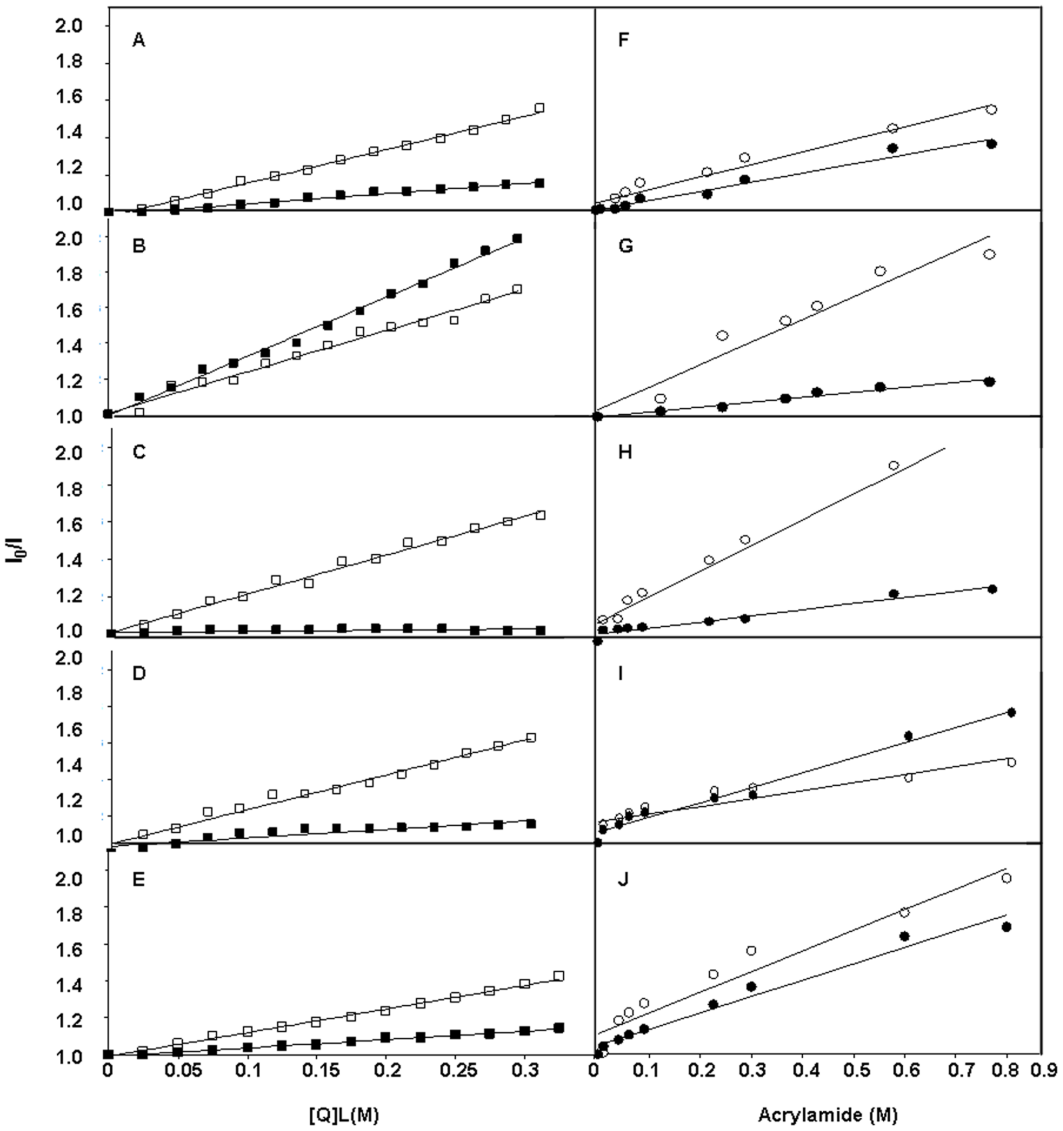
Localization of catechins within model membranes. Stern-Volmer plots of quenching of (A) ECg, (B) EC, (C) EC+ECg, (D) compound **3**, (E) compound **5** fluorescence with spin probes 5-NS (□) and 16-NS (▪) when incorporated into *S. aureus* model membranes. Quenching of (F) ECg, (G) EC, (H) ECg+EC, (I) compound **3**, (J) compound **5** fluorescence with acrylamide, either in solution (o) or incorporated into LPG:PG:CL vesicles (•).

Localization of catechins within the LPG:PG:CL membrane was examined with the quenching spin probes 5-NS and 16-NS, positioned within the fluid phase of the membrane. Stern–Volmer plots of fluorescence intensity changes following intercalation of catechins are shown in Figure 6. ECg fluorescence was quenched to a greater extent by the 16-NS probe, with the nitroxide group located deep within the bilayer, than by the more superficially located 5-NS probe. In contrast, EC fluorescence was more efficiently quenched by 5-NS compared to 16-NS, indicating that it occupies a more superficial location close to the lipid-water interface. These data complement previously reports of catechin localization within PC vesicles [14]. When EC and ECg were incorporated together into the model membranes in a 1∶1 ratio, the chromophore groups of both compounds were more efficiently quenched by 5-NS than by 16-NS, suggesting molecular rearrangement of membrane location when both compounds were present in the bilayer. Interestingly, **3** and **5** also adopted a location close to the lipid/water interface.

The hydrophilic quencher acrylamide, which preferentially quenches molecules in the aqueous phase or at the lipid/water interface [45], was employed to confirm the location of catechins in the bilayer (Figure 6). ECg was more efficiently quenched by acrylamide in buffer than in the membrane, indicating that this galloylated catechin is not accessible to acrylamide in the presence of phospholipids, providing further evidence for its location deep within the phospholipid palisade and corroborating results obtained with the spin probes. In contrast, EC was fully accessible to the quencher in both systems, supporting a superficial location for this compound. The fluorophores of both EC and ECg were more effectively quenched in buffer than in vesicles when both were present in the bilayer in a 1∶1 ratio. The combined spin label and acrylamide data suggests that, when incorporated within the same membrane, EC and ECg adopt a position close to the hydrocarbon chain-head group junction but not too shallow as to be accessible to hydrophilic quenchers. With **3** and **5**, there were significant differences in acrylamide quenching in buffer compared to vesicles, indicating a location comparable to that of EC and confirming the data obtained with the spin probes.

## Discussion

We previously established that exposure of logarithmic phase MRSA to ECg for the relatively long period of 4 h induced a substantial number of genes associated with the cell wall stress stimulon [26], a defensive response normally associated with bactericidal cell wall inhibitors [29]. The current study indicated that this response occurred within 1 h exposure to ECg and a number of genes encoding proteins involved in determination of the charge profile at the cell envelope were also up-regulated. In particular, those encoding enzymes involved with the attachment of D-alanine residues to WTA and MprF, the transferase responsible for substitution of PG head groups with lysine, complement biochemical studies showing that ECg reduced both D-alanine substitution of WTA [19] and lysinylation of PG [26] by 40–50%, eliciting a substantial increase in the overall negative charge of the staphylococcal surface and in all likelihood accounting for the phenotypic loss of capacity to form biofilms [8], [26]. MprF may modulate staphylococcal susceptibility to charged antibiotics, in particular positively charged antimicrobial peptides and DAP [67], [68]. Deletion of *mprF* from EMRSA-16 and a DAP-resistant mutant derived from this clinical isolate substantially increased the net negative surface charge but had no effect on oxacillin resistance in the presence or absence of ECg and the *mprF* gene product is, therefore, very unlikely to contribute towards ECg-determined increases in β-lactam susceptibility. Interestingly, and in contrast to studies with other *S. aureus* strains [69], neither Δ*mprF* mutant exhibited increased susceptibility to the polycyclic antibacterial peptide nisin.

ECg caused an immediate increase in the rigidity of the EMRSA-16 cytoplasmic membrane, but during subsequent incubation there was progressive reversion to a more fluid bilayer over 4 h (Figure 3A), reflecting substantial changes in lipid composition over this period [26]. Our studies with artificial vesicles modelled on the lipid composition of staphylococcal membranes indicated that the galloyl catechin showed a high affinity for LPG:PG:CL bilayers and interacted strongly with domains deep within the hydrophobic core. Interestingly, there appeared to be some organisation of the three lipid species into discrete domains within the artificial membranes, paralleling the existence of lipid microdomains in bacterial membranes [28], [70], and a degree of mixing of ECg and phospholipid within regions of the bilayer. This stable catechin-lipid association appeared to be sufficient to induce the changes in gene expression that we have observed in whole cells. In contrast, EC induced minimal changes to the fluidity of the staphylococcal bilayer, even after 4 h incubation (Figure 3B), and produced no significant perturbations of bacterial gene expression save for down-regulation of quinol oxidase genes, reflecting its superficial location within the bilayer at or near the water-lipid interface.

Our data has shed light on the capacity of EC to increase ECg-mediated reduction of staphylococcal oxacillin resistance over a wide range of EC and ECg concentrations [15]. Incorporation into the artificial bilayers of both molecules was enhanced following exposure to ECg and EC in combination. This combination had a more profound effect on the fluidity of the cytoplasmic membrane than either molecule alone and generated a greater degree of polarization of the DPH probe (Figure 3B), presumably because more ECg was present within the lipid palisade. Spin label and quenching data suggested that both molecules adopted a comparable location within the bilayer of artificial vesicles, in contrast to their behaviour when used as a single molecular species, to produce a higher degree of perturbation that accounts for their impact on membrane lipid behaviour as shown in Figure 3B. Thus, although it was not possible to distinguish the relative contributions of EC and ECg to fluorescence quenching when present in combination, there was a clear, dramatic change in their location within the bilayer. It is surprising, therefore, that both microarray and qRT-PCR data showed that the capacity of the EC-ECg combination to modulate EMRSA-16 gene expression was reduced compared to ECg alone; this is likely to indicate that the position adopted within the bilayer by ECg, in the absence of EC, maximizes the strength of the signals governing reconfiguration of cytoplasmic membrane architecture. Thus, although *vraX* was strongly up-regulated by the catechin combination as judged by qRT-PCR, it was significantly less than that found with ECg alone. Similarly, transcription of *dltA*, *mecA*, *fmtA* and *isaA* were significantly up-regulated by ECg but modulation of these genes failed to reach levels of statistical significance when cells were exposed simultaneously to ECg and EC (Table 2).

Differences in the capacity of ECg and EGCg to interact with lipid bilayers [14], [16], [17] suggested that changes to the B-ring hydroxylation pattern could influence the bioactivity of galloyl catechins. Compound **1**, with an unnatural 3,5-dihydroxylation of the B-ring, was indistinguishable from ECg with respect to oxacillin resistance modification but elicited a greater degree of up-regulation of some genes involved in the response to cell wall stress; in particular, transcription of *vraX* was greatly increased over control cells, twice as great as that for ECg (Table 2). There was also significant induction of *pbp2*, the foldase gene *prsA*, *sgtB* and *blaZ*, the gene encoding β-lactamase, implying a greater degree of membrane perturbation compared to ECg. In similar fashion, reduction of the B-ring substitution to a single hydroxyl function at the 3-position (Figure 1), yielding compound **2**, did not lead to increased resistance modifying activity but elicited enhanced modulation of some key genes over both ECg and compound **1**. For example, *vraX* was highly up-regulated and *agrA*, an accessory gene regulator that controls expression of a range of virulence determinants [55], was highly down-regulated over, suggesting that this molecule is likely to compromise virulence to an even greater extent than ECg [8], [54]. It was therefore surprising that **2** failed to induce “over-compensation” of membrane fluidity after 4 h incubation in a similar way to ECg and compounds **1** and **3** (Figure 3B); there appears to be no obvious reason for this highly reproducible finding as in other respects ECg, **1**, **2** and **3** represent a series of compounds with progressively enhanced membrane-perturbing properties. Although we have previously found little or no evidence that galloyl catechins modify the staphylococcal phenotype through direct interactions with cellular proteins [18], [26], it remains a possibility that the profound effect of **2** on virulence gene expression and relatively low impact on membrane fluidity is due to interactions with elements of the stress regulon or other two-component systems; this will be investigated in future studies. Compound **3**, carrying no hydroxyls on the B-ring, was associated with an increased oxacillin susceptibility-modifying capacity, and induced both a highly fluid staphylococcal cytoplasmic membrane comparable to the ECg/EC combination and a level of gene modulation higher than other molecules in this series, supporting the contention that progressive removal of –OH groups from the B-ring increased membrane interactions and bioactivity. In this context, **3** had a greater impact on the biophysical properties of LPG:PG:CL vesicles than ECg, strongly increasing the lipid order of fluid bilayers from a location close to the lipid/water interface.

Compounds **4** and **5**, in which all hydroxyl groups had been excluded from the A- and B-rings, possessed clear antibacterial activity and were poor resistance modifiers compared to ECg and analogs **1–3**. Compound **5** impacted primarily on EMRSA-16 by disrupting the integrity of the cytoplasmic membrane, reflected in the induction of a different transcriptomic profile compared to molecules **1–3**. The large majority of the genes selected for qRT-PCR analysis were significantly down-regulated at concentrations below the MIC, reflecting the overt antibacterial properties of this analog. Compound **5** dramatically altered the thermal characteristics of MLVs modelled on the *S. aureus* cytoplasmic membrane, shifting T_m_ to a lower temperature and creating a second peak of transition; **5** also appeared to render cells non-viable from a more superficial location in the membrane compared to ECg.

This study has shown that progressive removal of hydroxyl groups from ECg effects a transition from molecules eliciting an extremely weak antibacterial effect but characterized by a capacity to induce a β-lactam-susceptible phenotype to overtly antibacterial compounds with limited β-lactam-resistance modifying properties. All compounds in this series induced large increases in expression of the gene encoding VraX, a highly conserved 55-amino acid staphylococcal polypeptide of unknown function; *vraX* is exquisitely susceptible to up-regulation by agents interacting with the cytoplasmic membrane and its rate of transcription is substantially increased in the presence of cell wall-active antibiotics [48], [49]. This gene is under the control of the VraS/R regulator system and the genes encoding the response regulator (*vraR*) and the sensor histidine kinase (*vraS*) were also incrementally up-regulated in line with *vraX* expression. Deletion of *vraX* appears to have no detectable impact on the staphylococcal phenotype [71] and we have proposed, based on *in silico* modelling, that VraX is produced in order to sequester β-lactam agents before they are able to gain access to their target within the bacterial envelope [71]. In the current study, *vraX* was massively up-regulated by catechin gallates soon after exposure and would appear to be an early warning of lethal and non-lethal cell envelope perturbation. ECg is rapidly degraded *in vivo* due to the susceptibility of the ester linkage that joins the C-ring with the galloyl D-ring (Figure 1); a combination of some of the structural modifications described herein combined with substitution of an amide function for the degradable ester link could yield stable bioactive lead compounds with the capacity to alter the course of systemic staphylococcal infections when combined with conventional β-lactam antibiotics that have lost clinical utility due to the emergence of drug resistance.
